# Brief intervention content matters

**DOI:** 10.1111/dar.12044

**Published:** 2013-07-02

**Authors:** Jim McCambridge

**Affiliations:** Faculty of Public Health & Policy, London School of Hygiene & Tropical MedicineLondon, UK E-mail: jim.mccambridge@lshtm.ac.uk

For more than 30 years, there have been concerted efforts internationally to develop the evidence base for brief interventions in general practice 1. The choice of this setting reflected strategic judgements about where in health systems heavy drinking and alcohol problems were likely to be most encountered and thus where these interventions may be optimally delivered. This literature is usually interpreted along the lines that efficacy is now well established in general practice and that there is a need to extend study to new settings 2,3. This year has seen the publication of two large general practice cluster randomised effectiveness trials that in different ways draw attention to a crucial limitation of the evidence base in this setting; the lack of well-developed study of intervention content. Although both undertaken in the UK, they are likely to be seen internationally as important studies. The three-arm Screening and Intervention Programme for Sensible drinking (SIPS) trial compared a leaflet control condition against the same leaflet plus five minutes advice and the addition subsequently of 20 minutes counselling 4. Preventing disease through opportunistic, rapid engagement by primary care teams using behaviour change counselling (PRE-EMPT) compared training practitioners to address behaviour change for the big four key lifestyle risk factors (diet, exercise, smoking and alcohol) versus delayed training, thus entailing a non-intervention control condition 5. Both trials found no differences in alcohol outcomes for hazardous and harmful drinkers over a 12-month study period following interventions delivery by general practitioners and practice nurses.

The authors of these studies identify contrary implications for practice from these null findings. The SIPS authors conclude that the control condition: ‘screening followed by simple feedback and written information may be the most appropriate strategy to reduce hazardous and harmful drinking in primary care’ 4. One commentator, however, is concerned that this provides: ‘false reassurance that we have taken care of unhealthy alcohol use and will waste time and money’ 6 by delivering more time consuming interventions. In contrast, the PRE-EMPT authors, including myself, conclude that: ‘enduring behaviour change and improvements on biochemical and biometric measures are unlikely after a single routine consultation with a clinician trained in behaviour change counselling, without additional intervention’ 5. Attention to the detailed content of the evaluated interventions and consideration of their relationship to the existing general practice literature is necessary for interpreting study findings.

Following screening in SIPS, leaflet delivery was accompanied by the standard script reproduced in Box 1 (interested readers are strongly encouraged to examine the study website 7 for detailed information on all interventions). It will be seen that the feedback is very brief. The leaflet comprises content on health and social consequences, awareness of units (standard drinks), recommendations on daily consumption and where to get help. The advice intervention covers similar material providing additional tips on planning and the benefits of cutting down after being shown that drinking exceeded the majority of the population, without having further dedicated content on decision making, i.e. whether one should cut down 7. This was based on the Drink-Less material developed approximately 20 years ago, and in SIPS one hour training in delivery was given 7. The counselling intervention provides quite different material, being influenced by motivational interviewing, and requiring some understanding of this approach, though not proficiency in it 7. Counselling required a return visit to general practice, and just over half took this up 4.

**Box 1. The SIPS control condition**

‘Thank you for taking part in this project. Your screening test result shows that you're drinking alcohol above safe levels, which may be harmful to you. This leaflet describes the recommended levels for sensible drinking and the consequences for excessive drinking. Take time to read the leaflet. There are contact details on the back [Indicate where these are] should you need further help or advice’. [http://www.sips.iop.kcl.ac.uk/pil.php]

PRE-EMPT is a training trial that examined the effects of the ‘Talking Lifestyle’ training program on outcomes following a routine practice consultation. This program aims to enhance skills in undertaking behaviour change discussions 5. This intervention was thus not specific to alcohol, which may have been discussed only for those screening positive, most likely for those for whom this was their only positive screen. To see the detailed content of the training program visit http://www.3trials.net: login = guest10@cf.ac.uk, password = guest10, and then click on the Talking Lifestyles icon. Although there were good levels of engagement with the training program, skill acquisition was sub-optimal even though it was not designed for advanced practice. The listening skills that are a key feature of motivational interviewing were not included within the training program 5.

Approximately one-third of the trials included within the Cochrane primary care review compared a brief intervention with no intervention (as in PRE-EMPT), and in the other two-thirds the control conditions were the provision of leaflets or usual care in the form of general practitioner (GP) advice to cut down (as in SIPS) 8. This review incorporates some emergency room studies as well as those conducted in general practice. Overall evaluated brief interventions were found to be effective in reducing drinking by approximately 38 grams of alcohol per week more than the control conditions 8. This means that the findings from both SIPS and PRE-EMPT differ from previous studies in ways that remains to be explained, though the existing literature is not straightforward to interpret 9.

Both trials were pragmatic, intended by design to measure real world effectiveness, for which detailed investigation of intervention conduct could have interfered. The slow development of process study 10,11 has previously been widely recognised as a key weakness of this literature (see, e.g. 2) and the corollary to this is that we do not know as much about effective content as we should. As a result, there has been little or no evidence-based innovation in the design of advice interventions. In SIPS, there appears little difference in content between the evaluated leaflet and advice interventions other than in the extent of verbal interaction. Providing relatively patient-centred counselling after this type of advice 7 may not be optimal. As a training trial, the detailed content of the intervention was not the object of evaluation in PRE-EMPT, making it impossible to know how exactly these discussions were conducted 5. Counselling that simply calls upon the perspective of motivational interviewing, though does not implement it, as is the case in both trials, may simply not be helpful enough; non-specific forms of counselling do not have a good track record in this field (e.g. 12). Brief intervention content research questions are difficult to answer rigorously in pragmatic trials where effects may not survive well the translation into routine practice 6. This would be true even if the specific content has previously been established as efficacious, which it has not been for brief interventions.

It is an interesting possibility that leaflets with SIPS content accompanied by minimal verbal interaction can be effective in their own right and as effective as lengthier discussions, however unlikely this appears. Brief interventions in routine practice can be very brief 13, and this SIPS intervention deserves to be evaluated in well-designed randomised controlled trials that overcome barriers to interpretation cited by the SIPS authors, such as assessment effects 14. Saitz 6 suggests that the SIPS trial results are vulnerable to unhelpful interpretations and I suggest this is true of PRE-EMPT too. Applying findings from these studies directly to influence policy and practice is misguided, as they conflict with what was previously known in ways which call for better understanding of the literature as a whole. A leaflet and a few words about drinking may well be enough for some, though we have no idea who they are and this is not what the wider literature indicates.

It would be inadvisable to expect that anything delivered briefly as an intervention should be effective. There is reason to be concerned that many who might value the opportunity to talk about their drinking, and benefit from so doing, should not be denied that opportunity. It remains to be established which skills practitioners need to have, or how they need to be applied and for how long, in order to be able to discuss drinking or other behaviours in ways which do help people. Brief intervention content matters and we should study it.
